# Linkage disequilibrium pattern of the *ATM *gene in breast cancer patients and controls; association of SNPs and haplotypes to radio-sensitivity and post-lumpectomy local recurrence

**DOI:** 10.1186/1748-717X-2-25

**Published:** 2007-07-10

**Authors:** Hege Edvardsen, Toril Tefre, Laila Jansen, Phuong Vu, Bruce G Haffty, Sophie D Fosså, Vessela N Kristensen, Anne-Lise Børresen-Dale

**Affiliations:** 1 Department of Genetics, Institute for Cancer Research, Rikshospitalet-Radiumhospitalet Medical Centre, Oslo, Norway; 2 Department of Clinical Cancer Research, Rikshospitalet-Radiumhospitalet Medical Centre, Oslo, Norway; 3 Faculty of Medicine, University of Oslo, Oslo, Norway; 4 Biomedical Laboratory Sciences Program, Faculty of Health Science, Oslo University College, Oslo, Norway; 5 Department of Radiation Oncology, Robert Wood Johnson Medical School Associate, Cancer Institute of New Jersey, New Jersey, USA

## Abstract

**Background:**

The ATM protein is activated as a result of ionizing radiation, and genetic variants of the *ATM *gene may therefore affect the level of radiation-induced damage. Individuals heterozygous for *ATM *mutations have been reported to have an increased risk of malignancy, especially breast cancer.

**Materials and methods:**

Norwegian breast cancer patients (272) treated with radiation (252 of which were evaluated for radiation-induced adverse side effects), 95 Norwegian women with no known history of cancer and 95 American breast cancer patients treated with radiation (44 of which developed ipsilateral breast tumour recurrence, IBTR) were screened for sequence variations in all exons of the *ATM *gene as well as known intronic variants by denaturating high performance liquid chromatography (dHPLC) followed by sequencing to determine the nature of the variant.

**Results and Conclusion:**

A total of 56 variants were identified in the three materials combined. A borderline significant association with breast cancer risk was found for the 1229 T>C (Val>Ala) substitution in exon 11 (P-value 0.055) between the Norwegian controls and breast cancer patients as well as a borderline significant difference in haplotype distribution (P-value 0.06). Adverse side effects, such as: development of costal fractures and telangiectasias, subcutaneous and lung fibrosis, pleural thickening and atrophy were evaluated in the Norwegian patients. Significant associations were found for several of the identified variants such as rs1800058 (Leu > Phe) where a decrease in minor allele frequency was found with increasing level of adverse side effects for the clinical end-points pleural thickening and lung fibrosis, thus giving a protective effect. Overall our results indicate a role for variation in the *ATM *gene both for risk of developing breast cancer, and in radiation induced adverse side effects. No association could be found between risk of developing ipsilateral breast tumour recurrence and any of the sequence variants found in the American patient material.

## Background

The *ATM *gene was localized to the chromosomal sub-band 11q22-q23 by genetic linkage analysis in families with members affected by ataxia telangiectasia (AT) in 1988. AT is an inherited recessive disorder associated with neurological dysfunction, growth abnormalities, extreme radio-sensitivity, immunological deficiency and increased risk of malignancy [1-3]. The majority of AT- patients are compound heterozygous with different mutations in each allele of the gene, a large proportion of which are reported to be truncating, giving rise to shorter versions of the protein where the C-terminal domain of the protein often is deleted [4]. Individuals who are AT- heterozygous have been reported to have intermediate radio-sensitivity and an increased risk of malignancy, especially breast cancer [3,5-10], possibly associated with genetic variance affecting binding domains of the protein [11]. Estimates of carrier frequencies indicate that 0.5–1% of the population are AT-carriers [8,12]. Studies in mice have shown that *ATM *haploinsufficiency is followed by an increased sensitivity to low doses of radiation, carcinogens and an increased incidence of mammary tumours but not increased radiation mutagenesis [13-15].

The *ATM *gene codes for a protein with 3056 amino acids and a molecular weight of ~350 kDa which have been found to exist both in monomeric (active) and dimeric (inactive) state [16]. The protein contains several important domains such as ^1) ^the C-terminal protein kinase domain (PI3K-domain), ^2) ^the substrate binding domain in the N-terminal of the protein necessary for activation of p53 in response to DNA damage, ^3)^the FAT domain – common for the PI3K-like family members FRAP, ATM and TRAPP, ^4) ^a proline rich region shown to bind c-Abl and ^5) ^an incomplete leucine zipper. For more detailed description of the domains see the review by [17]. The protein is primarily located in the nucleus but has also been found in cytoplasmic vesicles called endosomes and peroxisomes. In the peroxisomes ATM co-localized with catalase which is involved in the detoxification of reactive oxygen species [18,19].

The ATM protein is involved in the cell cycle control and is a member of the phosphatidylinositol 3-kinase family, implicated in the early response to DNA damaging agents, such as ionizing radiation causing double strand breaks (DSB) [16,20]. ATM possesses kinase activity and phosphorylates serine and threonine amino acids in several important downstream cell cycle proteins such as p53, BRCA1/2, CHK1/2 and c-Abl [18,20,21]. ATM deficient cells are extremely sensitive to ionizing radiation (IR). It has been shown that IR induces the instantaneous phosphorylation of the ATM protein at Ser-1981 leading to catalytic activation by dimer dissociation rendering the kinase domain accessible [22]. This activation continues throughout the cell cycle although the protein level remains constant [23]. Recent studies have identified two additional serine residues, Ser-367 and Ser-1893 which are phosphorylated as a response to DNA damage *in vitro *and shown that site specific mutations of either one of the three serine residues (367, 1893 and 1981) give rise to proteins defective in ATM signalling *in vivo *[24]. Studies of linkage disequilibrium (LD) patterns of the ATM gene have revealed low recombination and extensive LD spanning the whole gene, in particular in the 3'- end of the gene, with few haplotypes representing the majority of chromosomes [25-27]. Studies of the associations of haplotypes with breast cancer risk have revealed contradictory results, some showing an increased risk associated with particular haplotypes [27,28] while other found no such association [29,30].

The aim of this study was to investigate the difference in type and frequencies of *ATM *variants and haplotypes in association with risk of breast cancer, as well as subcutaneous and cutaneous radiation induced adverse side effects, development of costal fractures and pleural thickening. In addition, we wanted to investigate whether an association between genetic variation of the *ATM *gene and the risk of developing local recurrence after radiation treatment could be found.

## Materials and methods

### Norwegian controls

The control group for the Norwegian breast cancer cases consisted of 95 post-menopausal women participating in the National Mammography screening program, with no history of breast cancer after two negative mammograms [31]; age range at the time of blood collection was 55 – 72 years.

### Norwegian breast cancer cases

The breast cancer cases used in this study has previously been investigated for variations in the glutathione-S-transferase genes *GSTP1*, *GSTM1 *and *GSTT1 *and are also described in detail in [32] as well as here: From 1975 to 1986 a total of 1496 patients diagnosed with breast cancer and referred to the Norwegian Radium Hospital (NRH), received their loco-regional radiation treatment with a fractionation pattern of 4.3 Gray (Gy) x10 (2 treatments per week for 5 weeks; total dose 43 Gy; treatment A). This fractionation schedule was applied both as an adjuvant, post-operative treatment and administered to women who had RT after a loco-regional recurrence following breast cancer surgery some years prior to referral to the NRH. This RT schedule was expected to be more effective and at the same time less resource-consuming than the conventional fractionation pattern (2 Gy x25, 5 treatments per week; total dose 50 Gy). The typical post-mastectomy target fields covered the ipsilateral lymph node regions in the axilla, the fossa supraclavicularis and along the arteria mammaria interna. Depending on the extent of the operation and/or the expected risk of local recurrence, the thoracic wall was also irradiated [33]. Late adverse effects are therefore expected in these anatomical regions, manifested as telangiectasias of the skin, subcutaneous fibrosis and atrophy, costal fractures, pleural thickening and lung fibrosis. During the late 80's and early 90's evidence accumulated for unjustifiably severe adverse side effects following this type of RT. In 1996 it was decided that all patients still alive (n = 289) should be systematically evaluated for radiation induced adverse effects within the target field as a basis to estimate the level of monetary compensation. A total of 245 patients took part in this evaluation.

In parallel with treatment A, an alternative treatment regimen was used (2.5 Gy x20, 4 treatments per week for 5 weeks; treatment B). This schedule still met the requirements related to limited RT capacity but was more in line with the conventional fractionation pattern of 5 weekly treatments of 2 Gy for 5 weeks. Treatment B was to be applied mainly in patients with primarily inoperable breast cancer, who could potentially be rendered operable by RT. From 1975 to 1991, 617 women received treatment B against the chest wall, with or without radiation to the regional lymph nodes. Of these 617 women, 155 were still alive in 1997. One-hundred-and-nineteen of these patients agreed to be included in the evaluation study and the same assessments of damage were performed as for the treatment A group.

During the survey, the clinical examinations and overall pain evaluation were performed by three dedicated oncologists. A physiotherapist assessed shoulder mobility and arm oedema by comparison with the contra lateral arm and also assessed the cutaneous and sub-cutaneous adverse effects. A radiologist recorded pleural and lung densities as seen on chest X-ray, in addition to the presence of costal fractures. Photographs of the irradiated areas were taken and kept in the patient's medical record. These photographs, together with the patient journals and the original evaluation, were the source of this study's scoring of cutaneous adverse effects, as assessed in 2004. All adverse effects were scored as "none", "little", "some" and "substantial", in part based on the CTC and Somalent scoring system and in part on an ad hoc defined scoring system based on the individual health professional's experience.

In the analysis of radiation-induced side effects we excluded patients who, after their primary RT, had repeated irradiations for loco-regional recurrence. As a result there were a total of 253 patients included with 156 having received 4.3 Gy x10 (A) and 97 having received 2.5 Gy x20 (B). Of these, 5 women (1 given treatment A and 4 given treatment B) had inoperable tumours and received the RT to shrink the tumour in order that they could receive surgery. The remaining 248 women (155 receiving treatment A and 93 treatment B) received post-operative RT [32].

### American breast cancer cases

The patients included in this study were part of a larger patient cohort containing a total of 1546 early stage breast cancer patients treated at Yale New Haven Hospital between 1973 and 1994 with lumpectomy followed by radiotherapy (LRT). A total of 112 patients developed ipsilateral breast tumour recurrence (IBTR), 52 of whom consented to participate in this study (group 1). As a control group, 52 women with breast cancer treated with LRT in the same period but not developing IBTR were collected (group 2). The two groups were matched by age (± 5 years), year of treatment (± 5 years) and stage of the disease [34]. Leukocyte DNA was available for all 104 samples but mutation screening of the *ATM *gene was only performed for 44 of the patients experiencing IBCT and 51 of the matched controls. This was done to fit into a 96 well format analyses scheme, excluding the samples with the poorest DNA quality and lowest DNA concentration.

### Consent form and ethical committee

All samples were collected after proper informed consent was obtained and the project was approved by the regional ethical committee.

### DNA isolation

Blood samples were collected in EDTA tubes and frozen until time of leukocyte DNA isolation using chloroform/phenol extraction followed by ethanol precipitation using the Applied Biosystems 340A Nucleic Acid Extractor and according to standard procedures.

### Genotyping Method

All individual exons of the ATM gene and some flanking intronic regions with known variants were screened for variants by denaturating high performance liquid chromatography (dHPLC). A thorough description of the method can be found in [35]. Briefly, individual exons and the included intronic regions were amplified by PCR and screened for variations performing heteroduplex analysis and separation on the Transgenomic^® ^Wave System. Heteroduplexes were identified by abnormal band pattern appearing on the chromatograms and samples with possible variations were subjected to direct sequencing of a newly amplified PCR fragment to determine the nature of the variant. Samples with a dHPLC band pattern deviating from the reference sequence, but without evidence for a heteroduplex were also submitted to direct sequencing in order to capture any homozygote variant. Both Wave and sequence output were read independently by two investigators. Sequence information on all PCR primers as well as the PCR and dHPLC conditions can be found in [35].

### Statistical Methods

To test the statistical significance of the difference in genotype distribution between two groups Chi-square tests were performed using SPSS 13.0. All p-values of single marker associations are two sided and not corrected for multiple testing. The haplotypes were estimated using Phase v.2.1.1 and the significance of the difference in haplotype distribution between two or more groups were obtained using the case-control permutation analysis implemented in Phase that tests whether the estimated haplotypes in the case and control groups are a random sample from a single set of haplotype frequencies or if cases are more similar to other cases than to controls [36,37].

### *In silico *protein analysis

The online protein prediction tool PolyPhen [38] was utilized to assess the possible functional effect of a sequence variation in the coding regions of the *ATM *gene resulting in an amino-acid substitution in the protein sequence. The online tool scores the effect of a non-synonymous variation as benign, possibly damaging or probably damaging.

## Results

**Figure 1:**
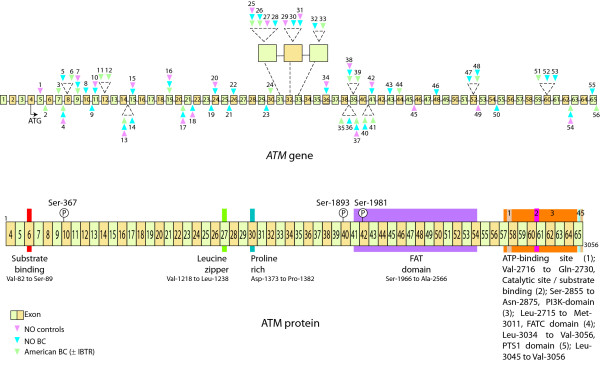
Schematic illustration of the *ATM *gene. The distribution of the variations detected in the studied materials along the gene is shown in the upper panel with exonic variants indicated on top of the gene and intronic below the gene, illustrated by colored triangles (pink for Norwegian controls, blue for Norwegian breast cancer patients and green for American breast cancer patients, numbers above/below is consistent with numbering used in Additional file 1). Below is given an illustration of the protein with important areas such as substrate binding domains, Leucine zipper, ATP-binding domains, FAT domain and PI3K domain [17] together with exonic information. (The size of the exons and the distance between them are not indicative of the sizes/distances in the gene/protein).

Screening of the exonic regions of the *ATM *gene, as well as known intronic variants, in three materials: Norwegian controls (material 1), Norwegian breast cancer cases (material 2) and American breast cancer cases, with or without ipsilateral breast tumour recurrence (material 3), identified a total of 56 variations; 55,4 % transitions (n = 31), 32,1 % transversions (n = 18) and 12,5 % insertions/deletions (n = 7), [see Additional file 1]. Of these, 10 were intronic and 36 exonic, the latter sub-grouped into: 3 truncating, 10 synonymous and 23 non-synonymous. Estimations of Hardy-Weinberg equilibrium were performed for the variants detected in the Norwegian controls, none of the variants deviated from Hardy-Weinberg equilibrium (data not shown). Nine of the 56 identified variants were found in all three materials, an additional 7 were common for the Norwegian materials (material 1 and material 2) and three for the breast cancer materials (material 2 and material 3). The variations were distributed throughout the gene, with the highest number of variants found in close proximity to or within exon 39 (5 variants), exon 31 (4 variants) and exon 8,15,32,52 and 60 (3 variants identified in each). The location of the identified variants along the gene as well as the exons relative to the domains of the protein described by [17], such as the PI3K domain, substrate binding domains and ATP-binding domains, is illustrated in Figure 1.

### Single marker associations

#### Risk for developing breast cancer

The association between any variant in the *ATM *gene and risk of breast cancer was computed by comparing the 95 cancer free women and the 272 breast cancer patients from Norway. A total of 43 variants were identified in the two materials combined [see Additional file 1]. The variant in exon 11 (variant nr. 10, Additional file 1), where a T to C substitution causes an amino-acid change from valine to alanine in the Leucine zipper domain, was found borderline significantly associated with risk of development of breast cancer (P = 0.055), with a lower frequency of the minor allele in breast cancer patients, suggesting a protective effect for of this variant (Table 1).

**Table 1:** 

**Exon/Variant**	**Genotype**	**Cases**	**Controls**	**P-value**
Exon 11				
1229 T>C	TT	270	92	0.055
Val > Ala	TC	1	3	

Variant associated at the single marker level with development of breast cancer in the Norwegian breast cancer patients

#### Association of variance in *ATM *with adverse side effects of radiotherapy

The impact of variation in the *ATM *gene on the level of radiation induced side effects: costal fractures, subcutaneous and lung fibrosis, pleural thickening, development of telangiectasias and atrophy, was studied in the Norwegian breast cancer patients. Twenty individuals were excluded from this analysis as a consequence of receiving multiple radiotherapy treatments to the same area, thus making it difficult to evaluate radiation induced damage from one specific treatment. The remaining 252 patients were first analyzed in combination (Table 2, a) and then divided according to treatment regimen and analyzed separately (Table 2b and 2c). A total of 154 and 94 patients received treatment A (4.3 Gy *10) and B (2.5 Gy *20) respectively. Several of the detected *ATM *variants were rare [see Additional file 1] and association analyses with level of radiation induced side effects were performed only for those with minor allele frequency > 1%. Even at this low frequency, several of the SNPs were found associated to one or more of the studied end-points: costal fractures, pleural thickening, subcutaneous and lung fibrosis, development of telangiectasias and atrophy both when all cases were analyzed in combination and when the cases were divided into two groups according to received treatment regimen (Table 2a,b and 2c). The change of a G with an A in exon 39 (rs1801516) was found significantly associated with the development of telangiectasias when all cases were analyzed combined (P-value 0.042), and the association became even more significant when only the patients receiving treatment A were analyzed (P-value 0.027). The association is caused by a decreasing frequency of the minor allele with increasing level of radiation induced side effects indicating a protective effect for the A allele. The C to T transition in exon 31 (rs1800058) altering the aminoacid in position 4258 from Leu to Phe was found associated with pleural thickening and lung fibrosis in all cases combined (P-value > 0.001 for both clinical end-points) as well as only in the patients receiving treatment B (P-value 0.001 and 0.002 respectively). Also in this patient group a borderline significant association was observed between this variant and development of costal fractures (P-value 0.055). The impact of this association and other listed in Table 2 have to be interpreted with caution since the number of identified variant alleles is very low and the number of cases limited.

**Table 2:** 

**Exon/Variant**	**Genotype**	**Level of adverse effects**				**P-value**
			
		**0**	**1**	**2**	**3**	
**a)**						
***Pleural thickening***						
Exon 20	GG	136	82	23	1	0.001
IVS20+28delG	GA	4	2	0	1	
Exon 31, rs1800058	CC	135	82	23	1	> 0.001
4258 C>T	CT	5	1	0	1	
Leu > Phe						
Exon 41, rs3092910						
5793 T>C	TT	136	82	23	1	0.001
Ala > Ala	TC	4	2	0	1	
***Lung fibrosis***						
Exon 31, rs1800058	CC	66	156	18	1	> 0.001
4258 C>T	CT	3	3	0	1	
Leu > Phe						
***Development of telangiectasias***						
Exon 39, rs1801516	GG	35	33	41	70	0.042
5557 G>A	GA	11	14	10	20	
Asp > Asn	AA	4	1	0	0	
***Atrophy***						
Exon 31, rs1800058						
4258 C>T	CC	35	57	74	65	0.02
Leu > Phe	CT	4	1	0	2	
**b)**						
***Pleural thickening***						
Exon 20	GG	69	61	19	0	> 0.001
IVS20+28delG	GA	2	2	0	1	
Exon 41, rs3092910	TT	69	61	19	0	> 0.001
5793 T>C	TC	2	2	0	1	
Ala > Ala						
***Lung fibrosis***						
Exon 32, rs1800889	CC	11	111	13	0	0.009
4578 C>T	CT	4	11	2	1	
Pro > Pro						
***Development of telangiectasias***						
Exon 39, rs1801516	GG	14	21	28	47	0.027
5557 G>A	GA	4	9	9	15	
Asp > Asn	AA	3	1	0	0	
**c)**						
***Costal fractures***						
Exon 9, rs3218674	CC	75	12	1	0	0.043
735 C>T	CT	6	0	1	0	
Val > Val						
***Pleural thickening***						
Exon 31, rs1800058	CC	64	20	4	0	0.001
4258 C>T	CT	5	0	0	1	
Leu > Phe						
***Lung fibrosis***						
Exon 31, rs1800058	CC	51	35	2	0	0.002
4258 C>T	CT	3	2	0	1	
Leu > Phe						
***Subcutaneous fibrosis***						
Exon 32, rs1800889	CC	32	25	16	5	0.022
4578 C>T	CT	6	1	0	3	
Pro > Pro						

Associations of genetic variance in the *ATM *gene with radiation induced side effects in the Norwegian breast cancer patients: for all patients combined (a), treatment A (4.3 Gy *10, b) and treatment B (2.5 Gy *25, c) (organized by adverse effect, level of adverse effect is divided into four groups: none (0), little (1), some (2) and substantial (3)). (The P-values are not adjusted for multiple testing)

#### Risk for ipsilateral breast tumour recurrence

None of the variants identified in the American breast cancer patients were found associated with risk of developing ipsilateral breast tumour recurrence (IBTR) at the single marker level although some differences in minor allele frequencies were seen (data not shown).

#### Association of heterozygosity of the *ATM *gene with adverse side effects of radiotherapy and risk for ipsilateral breast tumour recurrence (IBTR)

To assess the influence of variation in the *ATM *gene focusing on variants 1) affecting a splice site, 2) leading to a truncated version of the protein or 3) scored as probably or possibly damaging in PolyPhen, all patients with presence of one or more such sequence variation in the *ATM *gene where combined into one group. The level of adverse side effects in the Norwegian breast cancer patients or risk of IBTR in the American breast cancer cases were then compared between the group of patients with and the group of patients without any detected variation in the *ATM *gene fulfilling these criteria. No significant association could be found between the presence of such sequence variations in the *ATM *gene for any of the assessed end-points in the Norwegian breast cancer patients or the American breast cancer patients (data not shown).

### Haplotype associations

#### Risk for developing breast cancer

A trend for difference in frequency distribution of the haplotypes of the ATM gene was found between cases and controls when including all identified variants, (P-value 0.06) but it did not reach statistical significance. Phased estimations based on both cases and controls gave 51 haplotypes of which 12 were found in both cases and controls, 9 only in the controls and 30 only in the cases [see Additional file 2]. In addition, one haplotype was only found when analyzing the controls and another three only when analyzing the cases separately. The ten most frequent haplotypes were in common for both materials, and the top three accounted for 73.6%, 79.9% and 71.3% of the total number of represented chromosomes when analyzing cases and controls combined, only controls and only cases respectively. Calculating the difference in frequency distribution of the phased haplotypes, including only the variants with a minor allele frequency ≥ 1% in cases or controls, gave a P-value = 0.23. The low frequent variants tend to reside on different haplotypes.

#### Association with adverse side effects of radiotherapy

No significant association was found between haplotype distribution and the radiation induced adverse side effects studied here, whether the analyses were performed for all cases combined or split by treatment regimen (data not shown).

#### Risk for ipsilateral breast tumour recurrence

No significant difference in haplotype distribution was found in the American breast cancer cases with relation to risk of developing ipsilateral breast tumour recurrence. In both groups the three most frequent estimated haplotypes accounted for more than 78% of the analyzed chromosomes (data not shown).

## Discussion and conclusion

It has been reported that the coding regions of the *ATM *gene has a reduced nucleotide diversity in human and chimpanzee as compared to other genes such as *ABCB1*, *BRCA1/2*, *PTGS2 *and *XRCC1*, in particular the last 2650 bp of gene containing among other the PI3K domain [26]. Our results clearly illustrated this by the fact that only 11% of the total variation is found within this area. In addition, we see no variation in exon 6, which contains the substrate binding domain necessary for p53 activation. In a recent study of French AT-families [11] no difference in risk of breast cancer was detected between heterozygous truncating mutations and missense/in-frame deletions. Three high risk groups of truncating mutations were identified each of which were associated to a known binding domain of the ATM protein. Studying the association between sequence variations in the *ATM *gene and risk of breast cancer in seven family branches [39] found no association with mutations that truncate the ATM protein in these domains. This is in line with our results where the variant in exon 11 found associated with breast cancer risk is also not located in any of these domains. In a recently reported *in vitro *study the rs1800056 (Phe > Leu) and the rs1800057 (Pro > Arg) variants were found to modify chromosomal radiosensitivity in lymphoblastoid cell lines from AT-patients, AT-heterozygous and normal individuals [40]. These two variants were not associated with radiation induced adverse side effects in our study, but the rs1800058 (Leu > Phe), not found associated by [40] was linked to several of the clinical end-points analyzed here. [28] identified an association between the variant rs1801516 with radiosensistivity in French breast cancer patients caused by an overrepresentation of the A allele in the breast cancer cases who where adverse radiotherapy responders. This result is supported by the study of [41] where a trend towards increased radiosensitivity of human fibroblast where found with the presence of the variant genotype. This is in contrast with our results indicating a protective effect of the A allele. The contradictory between our study and that of [28] may be a consequence of the different ethnicity of the populations or possibly a result of the limited study population in the French study with only 70 radiosensitivity breast cancer cases included.

In accordance with recent studies we found that a small number of haplotypes represents the majority of the analyzed chromosomes [25,26], both in cases and controls. From a study of Korean breast cancer patients [42] reported a significantly different frequency distribution of the estimated haplotypes between cases and controls when analyzing five ATM SNPs with a minor allele frequency of more than 10%. None of the same variants were detected in our study as a consequence of both experimental design and the different populations studied but a trend indicating the same was found when analyzing our results although it did not reach statistical significance. Our data suggest that the low frequent variants are in part causing this difference.

Overall our results indicate a role for variation in the *ATM *gene both for risk of developing breast cancer, and in radiation induced adverse side effects, although the findings need to be confirmed in larger studies.

## Abbreviations

AT Ataxia telangiectasia

ATM Ataxia telangiectasia mutated

BRCA1/2 Breast cancer 1/2, early onset

CHK1/2 checkpoint homolog (S. pombe) 1/2

DSB Double strand breaks

FRAP FK506 binding protein 12-rapamycin associated protein (mTOR)

GST Glutathione-S-transferase

Gy Gray

IBTR Ipsilateral breast tumor recurrence

IR Ionizing radiation

kDa Kilo Dalton

LD Linkage disequilibrium

LRT lumpectomy followed by radiotherapy

p53 Tumor protein 53

PI3K Phosphoinositide-3 kinase

Rs Reference sequence

SNP Single Nucleotide polymorphism

TRAPP Transformation/transcription domain-associated protein, new gene symbol: TRRAP

## Competing interests

The author(s) declare that they have no competing interest.

## Authors' contributions

- HE, VNK and ALBD designed the study.

- TT, LJ and PV genotyped the samples from the American Breast cancer patients

- LJ and PV genotyped the samples from the Norwegian breast cancer patients and the Norwegian controls

- BH provided the samples from the American breast cancer patients as well as the clinical characteristics

- SDF collected the clinical characteristics of adverse side effects of treatment for the Norwegian breast cancer women.

- HE did the analysis of the results

- All authors have read and approved the final manuscript

## Supplementary Material

Additional data file 1 Overview of the variants detected in the materials investigated together with information on: position of variants in the genomic and cDNA sequence, predicted effect of aminoacid substitution by PolyPhen, rs-numbers, in which materials they were detected and the minor allele frequency of the variants in the different materials. Click here for file

Additional data file 2 The estimated halotypes from the case-control analysis of the Norwegian individuals with the number of chromosomes predicted to represent the different haplotypes in: cases, controls and cases and controls combined. Click here for file
